# Pancreatic transection due to blunt trauma

**DOI:** 10.4103/0974-2700.58657

**Published:** 2010

**Authors:** Amal Ankouz, Hicham Elbouhadouti, Jihane Lamrani, Khalid Ait Taleb, Abdelatif Louchi

**Affiliations:** Department of General Surgery, UH Hassan II of Fez, Morocco

**Keywords:** Pancreas, pseudocyst, transection, trauma

## Abstract

Blunt fractures of the pancreas are rare and serious lesions. An isolated injury to the pancreas is uncommon. Physical signs and laboratory parameters are often inaccurate, and missing the diagnosis can cause serious clinical problems. We report a case of a 28-year-old woman with blunt pancreatic trauma in whom computed tomography revealed a fracture through the tail of the pancreas. It was complicated by pseudocyst formation. She was treated surgically with good outcome. This case is a reminder that pancreatic injuries should be considered in the differential diagnosis in cases of blunt abdominal trauma. Also, the clinician should be aware that when pancreatic injuries are managed conservatively, the clinical, radiological, and laboratory parameters need to be monitored till resolution.

## INTRODUCTION

Pancreatic injuries occur in up to 10% of all major trauma events, with nearly 25% of these injuries resulting from blunt trauma. Due to the retroperitoneal location of the pancreas, isolated pancreatic injury occurs in less than 5% of cases of major blunt abdominal trauma.[1,2] Age, severity of injury, amylasis level, abdominal pain, injury severity score, presence of associated lesions, duration of shock, unrecognized diagnosis, and delay in treatment are the factors that significantly influence the outcome. The mortality is related to septic complications and subsequent pancreatic or biliary disruption. We herein describe a case of blunt abdominal trauma causing pancreatic rupture in a 28-year-old woman. The computed tomographic (CT) scan revealed a fracture through the tail of the pancreas. The patient was initially treated medically and developed a late pancreatic pseudocyst.

## CASE REPORT

**Figure 1:**
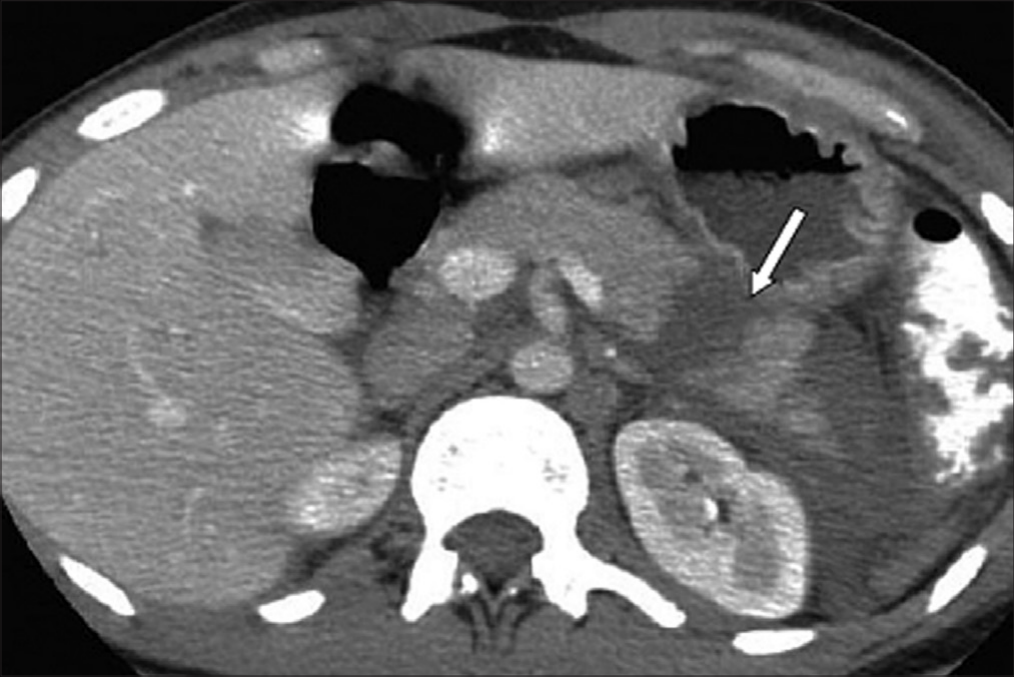
A computed tomography scan revealing a transection through the tail of the pancreas

A 28-year-old woman presented to the emergency department with epigastric pain of 6 days duration after an assault. She had received multiple blows to the abdomen but did not consult a doctor immediately, hoping that the pain would subside spontaneously. However, when the pain increased, she was brought to the emergency room by her family. On admission, she was stable (blood pressure: 120/70 mmHg) and mildly pyrexial (37.6°C). She was tender in her epigastrium. Investigations on admission revealed a white blood cell count of 9000/mm^3^, hemoglobin of 12 g/dl, and serum amylase of 800 IU/l. CT scan was performed [Figure 1] and revealed a transection through the tail of the pancreas (class II according to Lucas classification) [Table 1]; there was no injury to any other organ. As the patient was hemodynamically stable and there was no evidence of duct injury, she was treated conservatively (with bowel rest, nasogastric tube, and analgesia). She made a good recovery. Eight weeks later, she again complained of abdominal pain and vomiting. A CT scan was performed and showed a 7 cm pseudocyst in the epigastric region. A cystojejunostomy was performed and the patient was discharged 10 days after the operation.

**Table 1:** Modified Lucas classification of pancreatic injury

I	Simple superficial contusion or peripheral laceration, with minimal parenchymal damage; any portion of the pancreas can be affected, but main pancreatic duct is intact
II	Deep laceration, perforation, or transection of the neck, body, or tail of the pancreas, with or without pancreatic duct injury
III	Severe crush, perforation, or transection of the head of the pancreas, with or without pancreatic duct injury
IV	Combined pancreaticoduodenal injuries: (a) minor pancreatic injury, (b) severe pancreatic and also duct injury

## DISCUSSION

This case report shows that the diagnosis of a pancreatic fracture can be difficult at first. Trauma to the pancreas is not common, and isolated pancreatic trauma is even less common. The pancreas lies anterior to the vertebral column and may be compressed against it.[3–4] Injury to the pancreas is frequently combined with injuries to other organs, particularly the duodenum, and this may cause early death of the patient.[2,5,6] An isolated pancreatic injury may be missed or the diagnosis may be delayed because the initial symptoms and signs of pancreatic injury are subtle, and this may contribute to the morbidity and mortality associated with this injury.

Studies have demonstrated that the elevation of amylase in both serum and peritoneal lavage fluid is neither sensitive nor specific for the diagnosis of pancreatic injury.[7] Bradley, in a review of more than 400 cases reported in literature of blunt pancreatic injury, found that serum amylase levels were elevated in 82% of people with documented pancreatic injuries.[5] Because hyperamylasemia has been observed in more than 75% of patients with blunt abdominal trauma and proven pancreatic injury, it should at least be considered a sign of probable pancreatic injury in the setting of blunt abdominal trauma and should indicate the need for further testing.[5]

Helical multislice CT, which has both sensitivity and specificity as high as 80%, represents the best noninvasive diagnostic method for the detection of pancreatic injury. However, particularly in the initial phase, CT may miss or underestimate the severity of the damage; normal initial findings do not exclude pancreatic injury, and repeated CT in the light of continuing symptoms may improve its diagnostic efficiency.[8]

Morbidity and mortality rates for isolated pancreatic trauma are directly related to the presence of damage to the pancreatic duct. Preoperative endoscopic retrograde pancreatography is the only diagnostic test that has consistently shown a high specificity and sensitivity for pancreatic ductal injury. It is also valuable for planning the appropriate surgical correction (open surgery, internal transpancreatic duct stenting, or transductal drainage) for those patients who develop post-injury complications such as pseudocyst or distal chronic pancreatitis.[8]

Magnetic retrograde cholangiopancreatography was recently added to the list of useful pancreatic duct delineation techniques and could, in the future, replace endoscopic retrograde pancreatography as a first-line investigation, particularly with the development of rapid MRI imaging techniques.[9]

A nonoperative conservative course of management is common in pancreatic trauma. It is necessary to determine if there are signs of Wirsung duct injury and duodenal injury. In the absence of injury to the duct, close monitoring is done in a surgical unit. Medical treatment includes diet; rehydration, with correction of any electrolyte imbalance; nasogastric tube drainage in cases of vomiting; and analgesia. Preventive antibiotic therapy and octreotide are advocated.

If an injury of the Wirsung duct is likely or certain, treatment depends on the location of the pancreatic lesion. Acute endoscopic stenting of the disrupted main pancreatic duct has yielded excellent results in the hands of trained teams. Surgical intervention is usually undertaken in order to evaluate the pancreatic duct injury, to establish the presence of a devitalized pancreas, and to find out whether concomitant duodenal, biliary, or vascular injuries are present. Injuries of the pancreatic head are managed by external drainage, if there is no devitalization of the pancreatic head and if the duodenum and the ampulla are intact. In massively destructive lesions (with involvement of the pancreas, duodenum, and common bile duct) the decision to do a proximal duodenopancreatectomy is unavoidable. Injury to the neck, body, or tail of the pancreas with duct injury is best treated by distal pancreatectomy and splenectomy.

**Figure 2:**
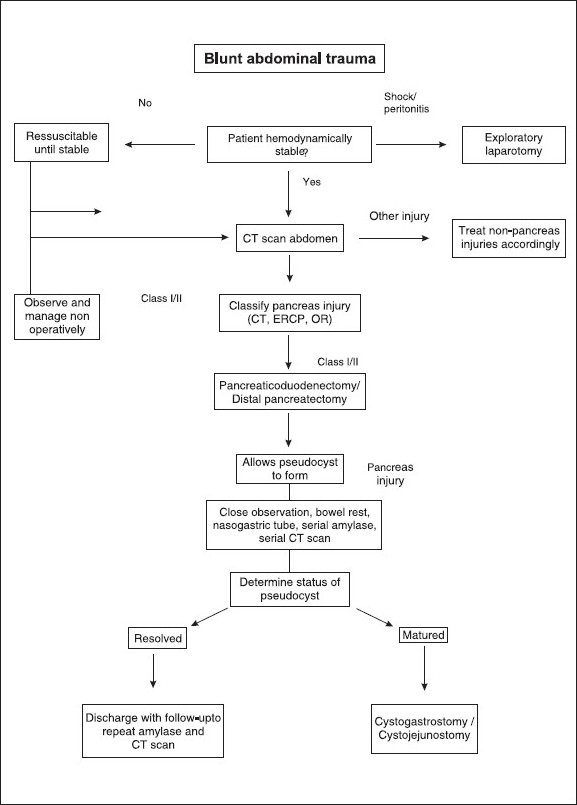
Algorithm for managing pancreatic injuries

Figure 2 represents a suggested algorithm for the treatment of pancreatic injuries after blunt abdominal trauma.[10] Among cases treated surgically for pancreatic trauma, 20–40% will present complications.[11] In the short term, sepsis and multiple organ failure cause 30% of deaths after pancreatic trauma.[8] After surgical treatment, secondary hemorrhage can originate from the pancreatic bed or the surrounding vessels as a result of retroperitoneal autodigestion The formation of pancreatic fistulas is common. The incidence of abscess formation ranges from 10–25%.[8] Mild pancreatitis may be anticipated in up to 18% of people who have undergone surgery for pancreatic trauma.[12] Endocrine and exocrine insufficiencies are very unusual after resection for pancreatic trauma. In the long term, pseudocyst formation can present weeks or months after the original injury.

## CONCLUSION

Suspecting a possible pancreatic injury after blunt abdominal trauma is important. When laparotomy is not required immediately, one can observe the patient and repeat the estimation of serum amylase. The use of modern contrast-enhanced CT, with or without 3D reconstruction, can usually clinch the diagnosis; however, if the diagnosis is still uncertain, emergency endoscopic retrograde cholangiopancreatography is advocated.
